# *Stevewoodia minutum*, a new genus and species of Scolytidae (Coleoptera) from the West Indies. Studies on West Indian Scolytidae (Coleoptera) 6

**DOI:** 10.3897/zookeys.56.517

**Published:** 2010-09-17

**Authors:** Donald E. Bright

**Affiliations:** Department of Bioagricultural Sciences and Pest Management, C. P. Gillette Museum of Arthropod Diversity, Colorado State University, Fort Collins, Colorado, 80527

**Keywords:** Curculionidae, Micracini, bark beetle, Caribbean

## Abstract

A new genus of Scolytidae (Coleoptera), Stevewoodia, from St. Lucia in the Lesser Antilles, is herein named and described. The type species, Stevewoodia minutum **sp. n.** is also named. The genus is named in honor of the late Steven L. Wood for his many contributions to the systematics of the Scolytidae.

## Introduction

The West Indies have been recognized as one of the world’s biodiversity “hotspots” (Mittermeier et al. 2005). This designation is based primarily on well-known groups such as vascular plants, mammals and birds. Since most insect groups are poorly documented, except perhaps butterflies, they are not generally considered in the usual biodiversity analysis; however, they probably constitute over 90% of the fauna.

A biodiversity study of the Scolytidae (Coleoptera) of the West Indies has been underway for a number of years and, for the past several years, I have been preparing a taxonomic monograph of the West Indian species of Scolytidae. While preparing that monograph, four specimens representing a new genus in the tribe Micracini were discovered.

## Systematics

### Stevewoodia gen. n.

urn:lsid:zoobank.org:act:31734B9E-0632-4122-971B-BB90DB3EA281

#### Diagnosis.

With the character states of Micracini (Wood 1982) but differs by the 5-segmented antennal funicle, by the solid antennal club with the sutures not visible, and by the very small and slender body which is 0.75–0.85 mm in length, 3.1 times longer than wide. Additional generic characters are included in the species description.

#### Type species:

Stevewoodia minutum Bright, sp. n.

#### Comments:

This genus is named in honor of the late Steven L. Wood, Monte L. Bean Life Science Museum, Brigham Young University, Provo, Utah, USA, the preeminent authority on the systematics and taxonomy of the Scolytidae. Dr. Wood was my major professor while I was a graduate student at Brigham Young University many years ago, and remained a valued colleague until his death in July 2009.

### Stevewoodia minutum sp. n.

urn:lsid:zoobank.org:act:92BC5FF3-F5AC-4AD6-88C7-3F8AE1B50353

Figs 1 2 

#### Materials examined.

**HOLOTYPE** (male), “WEST INDIES: St. Lucia, Mon Repos, 6.5 km N Fox Grove Inn, 10–28.VI.2007, submontane forest FIT, 300 m, S. & J. Peck”// “HOLOTYPE Stevewoodia minutum D. E. Bright, 2009.” **ALLOTYPE**, 1 (female), same data as holotype. **PARATYPES** (2); 1 (female), “WEST INDIES: St. Lucia, Bordelais trap site, 185 m, 13.9689N; 60.8859W, 05–09 JULY 2009, F.I.T., C. A. Maier, M. L. Gimmel & K. J. Hopp” and 1 (male), “WEST INDIES: St. Lucia, Bordelais trap site, 185 m, 13.9689N; 60.8859W, 10–-25 JUNE 2009, uv light, C. A. Maier & E. A. Ivie”

**Figure 1.**
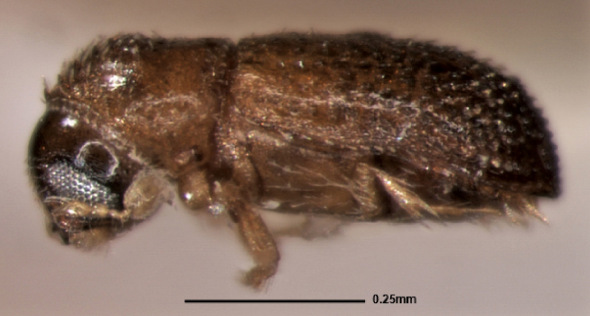
Stevewoodia minutum, lateral view of male.

**Figure 2.**
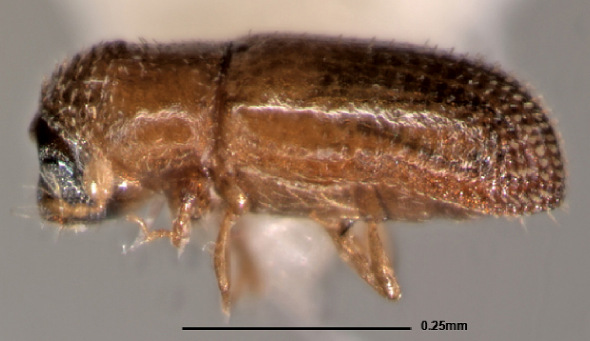
Stevewoodia minutum, lateral view of female.

#### Description.

The type specimens are presently in the author’s collection until the monograph is completed, at which time the holotype and allotype will be deposited in the Canadian National Collection of Insects, Ottawa, Ontario. The paratypes will be deposited in the collection of the West Indies Beetle Fauna Project at Montana State University, Bozeman.

##### Male.

Length 0.75 mm, 3.1 times longer than wide; light brown. Frons convex; surface moderately shining, densely minutely-reticulate. Antennal club oval, about 1.7 times longer than wide, solid, without sutures; scape as long as funicle, without obvious setae. Pronotum 1.1 times longer than wide, widest at base; sides weakly arcuate, anterior margin broadly rounded, with a few, fine serrations; anterior slope steeply convex, bearing scattered, small asperities, each asperity with a longer, flattened scale at posterior margin; summit slightly elevated; posterior portion weakly transversely impressed behind summit, surface shining, minutely reticulate, with scattered, obscure, fine punctures. Elytra 1.7 times longer than wide; sides parallel on basal three-fourths, strongly converging to narrowly rounded apex; discal striae not impressed, punctured in even rows, punctures very large, very weakly impressed; discal interstriae much narrower than striae, smooth, glabrous. Declivity convex; each interstriae bearing a median row of short, erect scales and very small granules. Protibia flattened, rectangular, with parallel sides; terminal mucro undivided. Procoxae contiguous.

##### Female.

Length 0.85 mm, 3.1 times longer than wide; light yellowish brown. Frons deeply concave from epistoma to well above upper eye level and laterally from eye to eye, lateral margin of concavity acutely margined opposite eye and acutely extended into a short elevation at upper margin; surface brightly shining, smooth, glabrous, with a clump of very short setae (barely visible at 96×) below elevation at upper margin. Antennal club as in male, except longitudinal line absent; scape as in male, except bearing long setae. Pronotum as in male except vestiture on anterior slope hair-like, obscure. Elytra and declivity as in male except declivital granules smaller, obscure.

#### Comments.

Adults of this species are easily distinguished from North American genera in the Micracini by their extremely small size, by the deeply concave female frons which bears a short, median elevation on the upper margin of the concavity, by the 5-segmented antennal funicle, by the narrow, elongate antennal club which is without visible sutures and by the presence of rows of erect scales on each declivital interstriae.

## Supplementary Material

XML Treatment for Stevewoodia

XML Treatment for Stevewoodia minutum
